# Editorial: Exercise prescription, physiological mechanisms, and exercise safety in patients with cardiac devices and arrhythmias

**DOI:** 10.3389/fphys.2026.1940884

**Published:** 2026-07-30

**Authors:** Caio V. Spaggiari, Maria Janieire de N. N. Alves, Giuseppe D’Antona

**Affiliations:** 1 Arrhythmias and Cardiac Pacing, and Exercise Physiology and Cardiac Rehabilitation Units, Instituto do Coração (InCor/HCFMUSP) – Hospital das Clínicas, da Faculdade de Medicina, Universidade de São Paulo, São Paulo, Brazil; 2 Centro di Ricerca Interdipartimentale Attività Motorie e Sportive (CRIAMS)-Sport Medicine Centre Voghera, University of Pavia, Voghera, Italy

**Keywords:** arrhythmias, cardiac implantable electronic devices (CIED), exercise prescription, exercise safety, physiological mechanisms

## Introduction

Cardiovascular diseases remain the leading cause of death worldwide, with cardiac arrhythmias contributing substantially to morbidity, mortality, and healthcare utilization. The intersection between exercise, arrhythmia management, and cardiac implantable electronic devices (CIEDs) has emerged as a rapidly evolving area of cardiovascular medicine, with important advances but persistent knowledge gaps regarding optimal prescription, mechanisms, and long-term safety. This Research Topic, Exercise Prescription, Physiological Mechanisms, and Exercise Safety in Patients with Cardiac Devices and Arrhythmias was conceived to address these gaps.

The studies in this Research Topic address three complementary questions: (1) how should exercise be prescribed for patients with arrhythmias and CIEDs; (2) which physiological mechanisms underlie its cardiovascular effects; and (3) how can exercise be prescribed safely in increasingly complex clinical populations? By synthesizing evidence across these pillars, the Research Topic provides a roadmap toward personalized, mechanism-informed, and technologically monitored interventions.

## Exercise prescription in patients with arrhythmias and cardiac implantable electronic devices

Exercise prescription has evolved from a conservative recommendation toward a personalized therapeutic strategy. Crisafulli et al. show that a 10-month individualized training program in a patient with painful left bundle branch block (LBBB) reduced resting heart rate by 15 bpm and raised the heart rate at LBBB onset by 50 bpm, dramatically expanding the safe exertional window. Dor-Haim and Davarashvili report exercise-induced normalization of chronic LBBB during recovery from stress echocardiography, underscoring the acute autonomic influence on conduction. In heart failure patients with right bundle branch block, Jing-Jing et al. demonstrate that conduction system pacing significantly improves QRS duration, ejection fraction, and functional class, supporting the concept that physiological pacing and structured exercise rehabilitation are complementary therapeutic strategies that may improve cardiac performance in these patients. Zeidan et al., in a retrospective analysis of 427 patients with paroxysmal supraventricular tachycardia, show that while first-line treatments are generally effective, comorbidities such as heart failure and ischemic disease predict poor response, reinforcing the need for individualized risk stratification when prescribing exercise and rehabilitation programs in this population. Collectively, these studies demonstrate that exercise prescription has evolved from a restrictive recommendation to a personalized therapeutic strategy capable of improving conduction, complementing device-based therapy, supporting safe physical activity, and enhancing cardiac performance.

## Physiological mechanisms underlying exercise responses

Understanding the mechanisms behind exercise-related benefits requires insight into autonomic regulation, electrolyte balance, and cellular adaptation. Li et al., in 1,556 university students, found that visceral adiposity correlates inversely with heart rate variability (HRV), linking metabolic state to autonomic tone—with pulse rate variability proving more sensitive than frequency-domain metrics for early detection. Wang et al. review potassium homeostasis, highlighting how disturbances in dietary intake, renal handling, skeletal muscle buffering, and ion channel function contribute to arrhythmogenesis and underscoring the need to monitor electrolytes during exercise, especially in at-risk patients. Zhang et al. review heat shock proteins (HSPs) in atrial fibrillation, showing they mitigate oxidative stress and remodeling; HSP levels may eventually guide exercise intensity and timing. Skoczyński et al. demonstrate that rapid atrial pacing evokes a robust parasympathetic response detectable via ultra-short HRV metrics (RMSSD and ΔPP), validating these measures for real-time autonomic assessment during exercise. Together, these studies form a continuum from autonomic regulation to electrolyte homeostasis, cellular stress, and real-time monitoring—providing a mechanistic framework for exercise prescription while highlighting the role of exercise in modulating autonomic regulation, adrenergic activity, and cardiovascular homeostasis.

## Exercise safety and emerging technologies

Safe exercise prescription increasingly depends on advanced monitoring. Hu et al. present a multi-channel seismocardiography system that captures exercise-induced shifts in aortic and mitral valve timing, enabling non-invasive assessment of cardiac mechanics during exertion. Liu et al. show that asmeton pretreatment significantly reduces dry cough and diaphragmatic contraction during pulsed-field ablation under conscious sedation—a finding that, while procedural, underscores the importance of patient comfort and safety. These technological innovations, combined with the device-related insights from Jing-Jing et al., point toward a future where wearable multi-channel seismocardiography systems, HRV monitoring, and emerging physiological sensors allow dynamic, real-time titration of exercise intensity.

## Future directions and conclusion

Despite these advances, key questions remain. What are the optimal exercise prescription parameters for specific arrhythmias and device types? Can biomarkers such as HSPs or potassium dynamics be integrated into routine care? What is the long-term safety of high-intensity exercise in ICD patients? Moreover, sex-based differences represent an important knowledge gap. Women may exhibit distinct physiological responses to exercise and could require lower exercise doses to achieve comparable cardiovascular benefits, underscoring the need for future studies to systematically evaluate sex as a moderator of exercise efficacy, safety, and prescription. Large, multicenter prospective studies will be essential to translate these observations into evidence-based clinical recommendations.

A recurring message from this Research Topic is that exercise prescription, mechanisms, and safety are interconnected components of precision cardiovascular care. Mechanistic insights inform individualized prescriptions, while monitoring technologies provide the safety framework to implement them in patients with arrhythmias and CIEDs. Collectively, these studies demonstrate that the three pillars are mutually reinforcing, and their integration is essential for translating science into practice.

The translational potential is substantial. Exercise-based interventions offer a scalable, cost-effective approach to improving cardiovascular health and reducing arrhythmia burden. We hope this Research Topic stimulates further investigation and contributes to a future in which exercise prescription becomes as evidence-based, precise, and personalized as pharmacological therapy.

We extend our gratitude to all authors, reviewers, and the Frontiers editorial team for their support.

